# *All Good Without Anything Good.* Beyond Survival: Understanding the Psychosocial Experiences of Individuals With Chronic Kidney Disease and Their Caregivers in Sri Lanka

**DOI:** 10.1111/hex.14157

**Published:** 2024-08-01

**Authors:** Darshika Thejani Bulathwatta, Agata Rudnik, Mariola Bidzan

**Affiliations:** ^1^ Department of Psychology and Counseling Faculty of Health Sciences, The Open University of Sri Lanka Colombo Sri Lanka; ^2^ Institute of Psychology Faculty of Social Sciences, University of Gdańsk Gdansk Poland; ^3^ Academic Center for Psychological Support University of Gdansk Gdansk Poland; ^4^ Institute of Pedagogy and Languages University of Applied Sciences in Elbląg Elbląg Poland

**Keywords:** caregivers, chronic kidney disease, culture, haemodialysis, health‐related quality of life, psychosocial experiences

## Abstract

**Aim:**

This study aims to explore the experiences of individuals with chronic kidney disease (CKD) undergoing haemodialysis and their caregivers, focusing on the disease's impact and the treatment process.

**Background:**

In Sri Lanka, CKD is a growing health concern, particularly affecting farming communities and contributing to the strain on the biomedical healthcare system. Despite increasing awareness of CKD's physical implications, its psychosocial impact remains underexplored. This study seeks to fill this gap, aiming to inform culturally sensitive interventions and improve the healthcare system's responsiveness to the unique needs of Sinhala Buddhist individuals with CKD and their caregivers.

**Design:**

An exploratory qualitative study.

**Method:**

Semistructured interviews were conducted with 10 individuals undergoing haemodialysis and 5 caregivers at a dialysis unit. The interviews were audio‐recorded, transcribed and analysed using conventional qualitative content analysis.

**Findings:**

The analysis revealed three interrelated main themes: (1) impact on standard of living (quality of life), (2) coping strategies and (3) medical experience, with a notable influence of traditional beliefs and practices.

**Conclusion:**

The findings highlight the need for a holistic approach to CKD management that integrates physical, emotional, psychological and social aspects, considering the significant role of traditional influences. Further research is essential to develop effective interventions that can enhance the quality of life for CKD.

**Patient or Public Contribution:**

The lived experiences of Sinhala Buddhist individuals with CKD and their caregivers served as a cornerstone, providing profound insights into the impact of the condition on their lives. Throughout the study, these participants played an instrumental role in refining the research's cultural sensitivity and relevance. Their engagement extended beyond the data collection phase to encompass feedback sessions, where they actively shared their perspectives. This ongoing collaboration ensured the study's depth and applicability to real‐world experiences. By actively involving those directly affected by CKD, this collaborative approach safeguards that the study remains rooted in their voices and addresses their unique needs.

**Reporting Method:**

This study adhered to relevant EQUATOR guidelines (the COREQ checklist).

**Trial Registration:**

This study is not a clinical trial, and thus, registration is not applicable.

## 1 Introduction

Chronic Kidney Disease (CKD) represents a significant global health challenge, impacting millions of individuals worldwide, including in Sri Lanka [1, 2]. Over the past three decades, the prevalence of CKD in Sri Lankan farming communities has notably increased, primarily due to two factors: the emergence of Chronic Kidney Disease of Unknown Aetiology (CKDu) and a rise in non‐communicable diseases such as hypertension and diabetes mellitus within the population [3].

CKD is defined as a decline in kidney function, characterized by a glomerular filtration rate (GFR) below 60 mL/min per 1.73 m^2^, or evidence of kidney damage that persists for at least 3 months, irrespective of the underlying cause [4]. The increasing prevalence of CKD is predominantly driven by hypertension and diabetes [5, 6].

As of 2017, the global incidence of CKD was reported to be increasing by approximately 8% annually [7], and it is listed among the top 20 causes of death worldwide [8]. Developing countries are experiencing a significant surge in end‐stage renal disease (ESRD), with a reported 70% increase, largely attributed to the high costs associated with treatment [9]. Moreover, in these nations, access to adequate dialysis facilities is often limited, which adversely affects survival rates for many individuals with CKD [10].

Abraham et al. [11] highlighted the impact of ethnicity on CKD prevalence, observing lower rates in white populations compared to Asians. They pinpointed South Asia as an ESRD ‘hotspot’, attributing this to several challenges, including limited access to healthcare, inadequate patient registries and deficiencies in early disease detection and management.

CKD typically progresses insidiously, often leading to the loss of up to 90% of kidney function before symptoms become apparent, eventually resulting in end‐stage kidney disease (ESKD) or ESRD [12]. According to the National Kidney Foundation's criteria (2002), CKD is categorized into five stages based on the degree of kidney function, with Stage 5 being ESRD. Conservative management is generally recommended through the earlier stages, but by Stage 5, when ESRD is reached, renal replacement therapies such as dialysis or kidney transplantation become necessary [13].

Haemodialysis, a primary treatment for CKD globally, presents significant challenges due to its demanding schedule, typically requiring lengthy sessions three times a week [14, 15]. While it extends life, haemodialysis often leads to a diminished quality of life for both patients and their families, affecting them physically, psychologically, and socially [16, 17]. ESRD, in particular, profoundly impacts various aspects of patients' lives, including familial relationships, education, finances, professional activities, physical health, social interactions and mental wellbeing [15].

Mckercher et al. [18] emphasize CKD as a global health threat that has not been adequately addressed. The journey of the disease is marked by disabling symptoms, dietary restrictions, social limitations and stigma, all of which significantly impact patients' lives [19]. CKD also adversely affects mental health, thereby influencing the overall Health‐Related Quality of Life (HRQOL) [20]. Research by Adejumo et al. [8] underscores the critical role of caregivers in patient care, highlighting that they often face unmet psychological needs. Notably, female caregivers are particularly susceptible to depression, anxiety and a sense of burden. Therefore, integrating supportive interventions for caregivers into treatment guidelines is essential to improve patient outcomes.

In Sri Lanka, the health sector, already under significant strain, faces potential cutbacks in critical services such as dialysis and transplants [21]. Senanayake et al. [20] indicate that Sri Lankan CKD patients experience high levels of psychological distress and depression. Ranasinghe and Ranasinghe [22] emphasize the necessity for enhanced psychosocial support, improved financial allowances and optimized resource utilization in the fight against CKD. Liyanage [23] advocates for an ethno‐medical approach, stressing the importance of cultural competency in healthcare to effectively bridge the gap between communities and hospitals. Furthermore, Wijayath [24] acknowledges the benefits of the global human rights framework in addressing CKD but points out that Sri Lanka's local capacity to deal with the disease is currently inadequate.

During the British colonial era, Sri Lanka's healthcare system underwent a significant transformation with the adoption of biomedicine, a shift that Mills [25] notes was largely insensitive to the country's traditional cultural practices. Liyanage [23] further critiques this approach, highlighting its neglect of the psychosocial and cultural dimensions of health risks. She emphasizes the need for more comprehensive research to understand the bio‐psychosocial needs of CKD patients, advocating for a more integrated approach in healthcare delivery.

The primary objective of this study is to delve into the psychosocial experiences and treatment journeys of individuals diagnosed with CKD and their caregivers. By gaining a deeper understanding of their unique bio‐psychosocial needs, the study aims to identify ways to more effectively integrate these needs into the current healthcare framework, thereby enhancing the overall quality of life for these individuals.

## 2 Materials and Methods

### 2.1 Procedure

This qualitative research is part of a broader mixed‐method study that investigates the psychosocial wellbeing of individuals with CKD undergoing haemodialysis, as well as their caregivers, across Sri Lanka and Poland. Before their participation, all participants received an informational document detailing the study's objectives, and their explicit consent was duly obtained. The data collection phase involved conducting semi‐structured interviews, based on prepared schedules, within the haemodialysis unit of the National Hospital, Kandy, Sri Lanka. The interviews were conducted by the principal investigator, an accomplished female researcher experienced in qualitative research, a university lecturer with an MSc and a psychologist. The data collection period spanned from 1 October to 31 October 2022. The research team's deep interest in the research topic, particularly the wellbeing of individuals with chronic diseases, motivated a nuanced exploration, ensuring a comprehensive understanding of the participants' experiences. All team members had backgrounds in psychology and/or psychotherapy, enabling them to offer appropriate debriefing and, when necessary, provide patients with psychological support. This study was reported using the Consolidated Criteria for Reporting Qualitative Research (COREQ) checklist [26] (File S1).

### 2.2 Participants

The study involved a total of 15 participants, comprising 10 patients with CKD and 5 caregivers of individuals suffering from CKD. Among these participants, there were 8 females (53%) and 7 males (47%), with an average age of 48 years. The demographic characteristics of the sample are presented in Tables 1 and 2. Purposive sampling, aimed at meeting specific research outcomes, was conducted in line with the criteria defined by Bulathwatta et al. [27]. The inclusion criteria for the study were individuals diagnosed with stage V CKD, currently undergoing haemodialysis treatment, aged between 18 and 70 years and having received CKD treatment for more than 6 months. Additionally, participants were selected based on nationality and religion, specifically those of Sinhala nationality and Buddhist faith. This study is part of a larger mixed‐method project aimed at comparing individuals of Sinhala nationality with Buddhist faith and Polish nationality with the Catholic faith. The inclusion criteria specifying Sinhala nationality and Buddhist faith were chosen for the Sri Lankan segment of the study to ensure cultural and religious homogeneity, which is crucial for the validity of the comparisons being made. By focusing on these specific criteria, we aim to control for cultural and religious variables, making it more convenient and consistent to gather data in Sri Lanka. This approach allows us to isolate the effects of nationality and religion on the study outcomes more effectively.

**Table 1:** Demographic characteristics of the participants (patients).

Participant code	Age (years)	Sex	Religion	Employment	Duration of the disease	Marital status
1	54	Female	Buddhist	Unemployed	2 years	Single
2	47	Male	Buddhist	Cemetery worker	9 months	Married
3	58	Male	Buddhist	Farmer	1 year	Married
4	61	Male	Buddhist	Unemployed	1 year	Married
5	59	Female	Buddhist	Retired Clerk	1 year	Married
6	57	Female	Buddhist	Traditional Sinhala Medicine practitioner	7 months	Married
7	39	Male	Buddhist	Unemployed	1 year	Single
8	24	Male	Buddhist	Unemployed	3 years	Single
9	45	Female	Buddhist	Unemployed	10 years	Single
10	58	Male	Buddhist	Retired Postman	1 year	Married

**Table 2:** Demographic characteristics of the participants (caregivers).

Participant code	Age (years)	Sex	Religion	Employment	Caregiver relationship	Months of caregiving	Marital status
11	23	Male	Buddhist	Unemployed	Son	1 year	Single
12	47	Female	Buddhist	Factory worker	Wife	9 months	Married
13	52	Female	Buddhist	Unemployed	Mother	3 years	Married
14	52	Female	Buddhist	Factory worker	Wife	1 year	Married
15	51	Female	Buddhist	Unemployed	Wife	1 year	Married

It is important to note that the presence of comorbidities was not considered in the inclusion criteria.

Conversely, the exclusion criteria for the study include individuals who do not meet the aforementioned inclusion criteria, those who are unwilling to provide consent and individuals who lack the physical or mental capacity to participate in the study.

For caregivers, the inclusion criteria include being identified by individuals with CKD as significant supporters, irrespective of the nature of their relationship (such as friend, spouse, child, sibling or parent), and being aged 18 years or older. Similar to the participants, caregivers who do not meet these inclusion criteria, who refuse to provide consent or who lack the necessary physical or mental capacity are excluded from the study.

### 2.3 Data Collection

The data collection phase consisted of face‐to‐face interviews conducted using semi‐structured interview schedules. These interviews, which ranged in duration from 35 to 60 min, were focused on exploring the participants' experiences with CKD. The process of data collection was continued until data saturation was reached, ensuring a comprehensive understanding of the participants' perspectives.

All interviews were audiotaped and initially transcribed verbatim in Sinhala. Subsequently, these transcripts were translated into English by an experienced researcher. To ensure accuracy and reliability, the English translations were then retranslated back into Sinhala. Independent researchers within the research team cross‐checked these translations to confirm that all nuances were accurately captured in the English version. For quality assurance, the transcribed materials were returned to the participants, allowing them the opportunity to comment and correction to ensure the fidelity of their narratives. Following this feedback loop, the finalized transcripts were utilized for the content analysis of the interviews.

### 2.4 Data Analysis

In this study, we employed an inductive approach, utilizing conventional content analysis to scrutinize the narratives derived from interviews, among other sources. This method entails a thorough examination of the content or contextual meaning within the text data, aiming to extract insights and deepen our understanding of the phenomenon being studied. We can characterize it as a systematic procedure of coding textual data to identify patterns, themes and relationships. Such analysis is highly versatile and can be applied to a wide range of written materials, regardless of the data collection techniques used. It is crucial in enhancing our grasp of individuals' perceptions and their lived experiences. Furthermore, the employment of open‐ended questions as an analytical tool significantly contributed to the depth of the study, aligning with a manifest analytical approach [28, 29].

Coding was carried out independently by two experienced researchers, each working to develop themes, categories and sub‐categories. This method, known as triangulation, aims to reduce potential biases that might arise from varying interpretations of the data by different researchers. In line with the guidelines proposed by Elo and Kyngäs [30] having at least two people encode the data independently is crucial for ensuring a more objective analysis. After completing their independent analyses, the researchers then convened to finalize the organization of the data and to reach a consensus on their findings.

### 2.5 Ethical Considerations

All participants were provided with comprehensive information regarding the scope and procedures of the study. Participation was entirely voluntary, and they were assured that they could withdraw at any time without any repercussions. To ensure confidentiality, each participant was assigned a unique encrypted identifier, guaranteeing their anonymity. Access to the collected data was strictly limited to members of the research team. The study received ethical approval from the Ethical Review Committee of the Open University of Sri Lanka, under the application number ER/2022/007.

## 3 Findings

**Figure 1:**
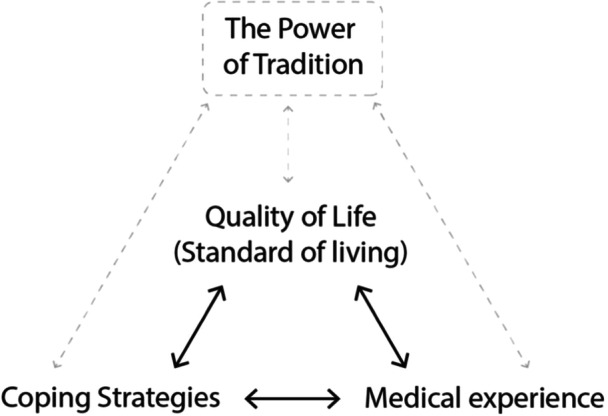
A model illustrating the mutual relationship among three identified themes and the influence of the power of tradition on them.

After analyzing data from individuals with CKD and their caregivers, three main themes emerged: (a) standard of living (quality of life), (b) coping strategies and (c) medical experience (refer to Table 3). Notably, these themes were found to be mutually interconnected. Interestingly, the ‘power of tradition’ was observed to influence all three themes (see Figure 1).

**Table 3:** Themes, categories and subcategories that emerged from the data.

Themes	Categories	Subcategories	Supported evidence/narratives
Standard of living (quality of life)	Work‐life	Looking for a job	Participant 8, despite having qualified for university through a competitive examination, was compelled to discontinue their education owing to the challenges posed by CKD and the demands of haemodialysis treatment. Additionally, his health condition significantly hindered his ability to find employment. *Beyond these challenges, my ability to focus on education has been severely impacted, and I deeply miss engaging in my studies. The nature of my condition prevents me from undertaking strenuous work. Additionally, I frequently experience fatigue, which significantly affects my capacity to work at the same level as others. While I am capable of working, the necessity of undergoing dialysis twice a week makes maintaining regular employment unfeasible. Taking two days off every week is not something I can afford.* Participant 11, a 23‐year‐old son and a caregiver to his father, explained the difficulty of finding a job because he has to take care of his father*.* *In his words, ‘I have so many challenges. Useless to tell (He was smiling). This is the time I should be starting my life. I have many challenges such as earning money, building a house, buying a car—everything. I do not have anything. I have to start from zero*’.
		Difficulties in keeping the job	Participant 1, who was formerly a practitioner of traditional Sinhala medicine, encountered significant challenges in transitioning to a life with dialysis. The demands of the dialysis treatment led to the closure of her shop, where she previously sold traditional remedies. Now, she has adapted by offering treatments at her home to those who visit her. *I lost my job, and life has become more difficult since I could no longer work. Managing expenses became challenging; for instance, I had to pay Rs. 20,000* [$ 62] in *rent for my shop per month, along with electricity bills, which led me to close it. Now, if someone seeks treatment, I provide it at home. However, the cost of bus fares to get to my treatments is high. I need to dedicate around 20 days each month to treatment and tests, which is quite stressful. This situation was a key factor in my decision to close the shop. As a result, my income has significantly decreased.* Participant 2 expressed that his wife stopped the job as she has to take care of him. *My wife stopped going to work after working for about 11 or 12 years. She stopped because of my condition. I have been requesting her to go back, but she isn*'*t listening to me. For the past week, she has been firmly telling me that she will not go back to work. She is refusing because she wants to take care of me.*
		Losing the job	Participant 5, along with her husband, resigned from their jobs due to the demands of her illness and treatments. As she explained; *I did not retire. I faced many obstacles at once, so I resigned. First, I had some difficulties such as a poor appetite and vomiting. I also had high blood sugar. But no one told me that I had a problem with my kidneys. It was only at this hospital that I found out about my kidney problem. Since then, I have been coming here for medications and treatments.* Her husband, who has been an excellent caretaker, took on the responsibility of managing all household chores effectively. *He limits his outings to only essential errands, like buying groceries and medicine. Apart from that, he is always by my side, assuring me to call him whenever I need anything. He was previously employed at the electricity board and had the option to continue working there until the age of 65. Yet, due to my illness, he felt compelled to resign as well, since there was no one else available to assist me. He became the one to take me to the hospital for my treatments* [Her voice conveyed a sense of depression as she spoke].
	Family life	Intimacy	Participant 4 explained his wife's response once he was diagnosed with the disease. *We*'*ll get through this together. We*'*ll adjust our diet, and prioritize your medications, and I*'*ll be here to support you every step of the way. We*'*ll face this challenge as a team. And remember, we have a wonderful support system with our family and friends too.*
		Isolation	Participant 1 [unmarried] expressed this: *They also feel sorry for me. One sibling lives in Mathara* [a district in Sri Lanka] *and the other in Seeduwa* [a city in Sri Lanka]. *They have young children. I have another sister in Gampaha* [a district in Sri Lanka]. *who calls me, but it*'*s hard for her to visit frequently When I was healthier, I supported my siblings*' *kids by having them stay with me. I did this without expecting anything in return. If I can help now, I still will.* participant 9 [unmarried] explained: *They* [siblings] *face their own difficulties. They*'*ve made mistakes, but what can be done? They have children, and they need to take care of them. So, I don't hold their mistakes against them.* Participant 3 shared feelings of detachment from his siblings, a contrast to the significant support he had provided them before he fell ill. *I have two younger brothers who live nearby; one resides just half a kilometer away, and the other is even closer. Yet, despite the short distance of less than 1 kilometer, they haven't visited me since I became ill. This is in stark contrast to our previously close relationship, as they have distanced themselves from me after my illness.*
		Impact on family members	Participant 8 explained the impact of his disease on his siblings. *They are very upset and depressed. I was selected for the university as well, which makes them sad as I cannot attend. The only relief is going for a transplant, so they are trying to arrange a transplant* Participant 13 described the impact on her family when her son was diagnosed with CKD and began dialysis. *Following my son's health crisis, my husband was also diagnosed with high blood pressure. Initially, he had intended to donate his kidney to our son. Unfortunately, he, too, was later diagnosed with diabetes and high blood pressure, conditions he had never experienced before. His health deteriorated due to the mental strain caused by our son's illness. The bond between my husband and our son is profound. Whenever our son's condition worsens, my husband becomes anxious and exhibits symptoms akin to a heart attack.* Participant 12 emphasized her role in supporting her husband through the challenges of the dialysis process. *It's a challenge for my husband to handle the dialysis on his own due to his condition, and it's not practical for me to depend on others to accompany him. He has a strong preference for either me or our son to be with him during these times. While I can't ask my husband to forgo his treatments, it's also important for both my son and me to focus on our futures. My son needs to carve out his own path, secure a job, and not be confined to staying at home to care for his father.*
	Everyday life	Diet	Participant 1 shared that her financial constraints made it difficult for her to afford the diet recommended by the hospital: *To be honest, affording the diet recommended by the hospital is beyond my means. I simply don't have the financial resources to purchase protein‐rich foods like meat, eggs, or milk. Currently, I weigh only 45 kg, and the hospital advises a daily intake of 40–50 grams of protein. Unfortunately, meeting this requirement is just not feasible for me.* Participant 7 mentioned the adherence to the hospital's prescribed timetable and strict dietary regimen, abstaining from unnecessary food items like tea and outside meals: *I have fully embraced the hospital's prescribed diet plan. I maintain a stringent adherence to both my dietary requirements and medication regimen, consciously avoiding unnecessary indulgences such as tea and dining out.*
		Financial issues	Participant 2 mentioned that his job is not sufficient to cover his medical and travel expenses. According to his words: *The current situation in the country is not good, so I fear my job won't be enough*. [The patient was emotional]. *We anticipate spending more than RS 1500* [approximately $5] *on travel expenses for the treatment.* Participant 5 explained her financial difficulties in her treatment process. *We have financial problems. We do not have a pension because we were working on a board. We spent all the money we saved on building a house and on my treatment process. Now our financial situation is zero. Without spending money, how can I recover myself?* *I also have to spend money on transportation. I come by a hired three‐wheeler for dialysis.* Participant 11 explained the expenses for drugs and the need for government revisions in selecting criteria for allowances. *Due to the economic crisis in the country, there is a shortage of drugs here. Some drugs are not available locally, so we have to buy them from outside. There is a specific drug needed for dialysis. We were advised to purchase it from outside, which costs about RS 1600 (6 $). I met a person who works as a development officer in his area. He said that he could get an allowance of RS. 20,000 (66 $). However, people like my father only receive a pension. The government should take action for people like my father. I think it would be better if there were a program for patients who have retired. They can properly analyze the situation and help people like my father. I do not expect a large amount. Some amount can be used for traveling for treatment, etc.*
		Social isolation	Participant 9 expressed her experience: *I can't hang out with friends because they're either busy with work or have moved away. Additionally, some people avoid me because of my sickness. This includes even some family members, who fear that my illness might cause problems for them. It's challenging because my condition isn't something that goes away quickly, like a cold. As a result, some people keep their distance. It's disheartening to witness this in our society—where sick individuals like myself are left out. It feels unjust.*
		Adaptation	Participant 1 shared her adaptation to the disease due to prolonged suffering. She remarked: *I didn't find out about my condition all at once, so it didn't shock me. I learned about it gradually, which helped me prepare mentally. Yes, I've been dealing with diabetes for a long time; I found out when I was 23.* Participant 14 disclosed her emotional turmoil, saying: *I was utterly shocked and mentally distressed. I lack the words to fully articulate it. Yes, at times, I experience anger easily. Sometimes, I lose my temper. But I have to consider my children, so I strive to maintain a calm state of mind.*
Coping strategies	Attitudes	Helplessness and hopelessness	Participant 3 explained that he feels helpless as he does not have children and no one is there to look after his wife if something happens to him. *It's hard, and sometimes the helplessness is overwhelming, but we have to keep going.* Participant 9, who had worked abroad before getting this disease, explained her helplessness due to the lack of social support. *I think they may believe they will face troubles because of me. This is trouble. It is not cured. This condition is not going to be cured like a cold or a fever. It exists forever. That is why they separate from us. This is a wrong thing that is happening in our society. People maintain a distance from us.* Participant 2 elucidated the difficulties in finding a suitable kidney for transplantation, stating: *I believe that if I find a donor, it should ideally be a family member, as external individuals may not be as inclined to donate. Additionally, being blood group O+ makes it more challenging to find a compatible kidney. Consequently, I've decided to continue with treatments like dialysis, while resigning myself to the possibility of a shorter life expectancy. Now, at 47 years old, the future seems uncertain. I am aware that beyond the age of 50, our physical strength tends to decline significantly.* Participant 3 explained that he feels helpless as he does not have children and no one is there to look after his wife if something happens to him. *I cannot explain, miss. I was so sad. We both do not have anyone else, and it felt like the world was falling apart.*
		Being focused on finding solutions	Participant 5 highlighted that her husband had assumed all household responsibilities while caring for her, due to the lack of external assistance. She explained: *My husband expressed that hiring help for me would incur expenses we currently cannot afford. Consequently, he manages all household chores, including cooking, washing, and cleaning. He diligently takes care of the tasks I used to handle, like washing clothes, preparing meals, and more.* Participant 3 explained that he sold a house to deposit money for their future expenses. *I did not have money when I got this disease. All the jewelry was in a bank. I had land with a house. As I was farming, I built another house. So I wanted to sell that house and the land. No one was buying. But, before 4 months, I could manage to sell it. Now I have 30 lakhs in my bank. Now we are living with the interest on that amount. I will get about 60000 every three months. So, it is enough to have a normal life.*
	Psychosocial support	Relatives' support	Participant 8 explained how he received family support, stating: *They only prepare appropriate food for me. My family's food pattern has completely changed due to my disease. They've shifted to eating more fruits and using less oil in cooking. They make sure that any food they bring home is good for me. Everyone has adapted to this. My sister, a midwife, also helps me a lot, even though she lives separately after getting married. She regularly visits and assists me in various ways.* In contrast, Participant 3, who has no children and no support from siblings, expressed deep sadness, saying: *I can't quite put it into words. I've felt a profound sadness. My wife and I are alone. Over time, I've adjusted somewhat.*
		Friendship	Participant 4 explained the support he receives from his friends, saying: *I frequently meet with my friends and talk to them. They have been a great help to me, even in my search for a kidney. They make sure I'm never alone when I come home; someone always accompanies me and ensures I am safely dropped off.* Participant 9 has a different experience with friends: *I can't hang out with friends because they're either busy with work or have moved away.*
		Government support	Participant 7 stated: *I receive Rs 5,000* [about $ 15] p*er month from the government. Additionally, my friends are also helping me*. On the other hand, Participant 8 mentioned his lack of government support despite being qualified. He said: *I did not receive anything from the government, even though some patients are receiving Rs. 5,000. My mother inquired about this with the GS officer* [a government officer who works for the village], *who refused it because we own a truck. The hospital recommended me for this support, saying I could get Rs 5,000 as all patients should, but I still did not receive it. So, I have not received any assistance from the government.*
	Spirituality/religion	Religious and cultural practices	Participant 6 expressed gratitude for her recovery and vowed to visit spiritual places to fulfill promises made to God. She said: *I've experienced immense benefits through cultural rituals. My daughter prayed for my health at the Temple of the Tooth, and I recovered. In gratitude, she gifted me a pendant for divine protection. I've made numerous promises to various spiritual places due to my recovery and am planning a journey to Ruwanwelisaya* [a stupa in Anuradhapura]. Participant 14 also mentioned finding relief by attending Bodhi Puja: *I regularly attend Bodhi Puja, and although my family doesn't join as often, they do participate during significant events like Katina* [the offering of new robes to Buddhist priests]. Participant 1 discussed her reliance on Buddhist philosophy, finding peace in life's impermanence through meditation, as she explained: *I find relief in Buddhism. Its teachings on impermanence resonate with me. Through meditation, I constantly remind myself of this, helping me to relax.* Participant 5 shared her perspective on embracing Buddhism until death, stating: *I aim to live without troubling anyone. Contemplating my Buddhist faith helps make my pain vanish. Dying with a spiritual mindset causes no trouble to others.* Participant 13 finds solace in meditation, a core teaching of Buddhism that helps him endure suffering: *Meditation is my solace against suffering. I've lived righteously and wish for my son's recovery.*
		Meaning of life	Participant 2 was trying to understand the psychological and physical aspects of the disease condition through the core Buddhist belief of ‘letting go’. *The solution is to practice letting go. Then we do not have problems* Participant 10 explained suffering is a compulsory thing in life. *I think the whole life is suffering.* Participant 4 expressed a serene acceptance of life's limitations, stating*:* *I'm not overly concerned. I'm 61 now. What's the point in expecting a longer life?*
	Perception of the disease	Cause of the disease	Participant 1 reflected on her efforts to protect herself, saying: *Since 2009, I've tried to protect myself. But perhaps this is my destiny.* Participant 7 shared his thoughts on life and unfulfilled desires, stating: *At my age, people usually marry and have children. I long for that life, and sometimes, I feel sorrow. It's like I've committed past wrongs (paw), and this feels like a punishment for something I did in a previous birth. I mourn the life I've missed—especially playing with kids. It feels like a consequence of past sins.* Participant 9 contextualized her current situation with a perspective on fate: *I see this as my fate, possibly due to misdeeds in a previous life. I acknowledge that others face even greater challenges. Some endure sudden losses, like limbs, or even their lives in accidents. I still have functional limbs and can earn a living, which brings me happiness. But ultimately, we all face mortality.* Participant 6 prompted contemplation and connected her disease with the Buddhist concept of karma, observing: *None of my family members have this disease. It seems I alone have this karmic disease* [‘karma lede’ in Sinhala].
		Better to live with fewer expectations	Participant 4 expressed a serene acceptance of life's limitations, stating: *I'm not overly concerned. I'm 61 now. What's the point in expecting a longer life?*
Medical experience	Process of diagnosis	Examinations	Participants undergo continuous check‐ups until their transplantation. As Participant 9 elaborated on her transplantation process: *I asked a doctor, who mentioned it might be possible, but I'll need to consult with a surgeon. The surgeon is the one who decides if I'm a suitable candidate. After that, a consultant will refer me for a transplant.*
		Insecurity	Participant 13 shared her experience when her son unexpectedly began dialysis, saying: *The doctors informed me that they initiated dialysis due to my son's elevated creatinine levels and revealed that his kidneys had been damaged. We were completely unaware of this disease. When we inquired about treatments, they provided no information.*
		Other chronic diseases	Participant 7 mentioned his long history with diabetes. He stated: *I have been suffering from diabetes since I was 18 years old.* Participant 4 also shared his health struggles, stating: *I have been treated for diabetes and hypertension for a long time*
	Gaining knowledge	Looking for information	Participant 1 expressed frustration with doctors who appeared reluctant to provide information beyond prescribing medication, leaving patients with a limited understanding of the disease. She explained: *Doctors aren't open to answering our questions. They seem unwilling to engage beyond just medication. As a result, we lack comprehensive knowledge about the disease. The nurse only advised on dietary changes in the later stages, when my creatinine levels increased. Before that, clinic visits were just about blood and urine tests with no additional advice. I was under their care for a long time but received no earlier guidance.*
		Health literacy	Participant 7 admitted his confusion about the disease, citing the varying information he received. He said: *I'm not sure what this disease exactly is. Some say the kidneys are melting; others say they're shrinking.* Participant 3, who was unaware of the effects of dialysis, questioned its impact, stating: *I don't even know what dialysis is, even though I'm undergoing it. Does dialysis make people weak?*
	Treatment	Difficulties with travelling	Participant 11, a caregiver for his father undergoing dialysis, detailed the challenges they faced due to long‐distance travel. He described the post‐dialysis routine, saying: *There is a dialysis shift from 7 p.m. to 12 a.m. Patients arriving for that shift, especially those from distant places, can't leave immediately; they stay overnight. We often end up sleeping on chairs or even on the floor. Many of us manage somehow, with some laying cardboard for comfort. You can see how people make sleeping arrangements. There's a bus from Colombo to Nuwara Eliya, arriving in Kandy around 3 a.m. We could take that bus and get off at Walapane. From there, another bus takes us to our village, reaching home by 7:30 a.m. However, that bus is incredibly crowded, making it difficult to take my father. As a result, we're compelled to stay until morning. Many others are in the same situation, making makeshift arrangements to sleep. As I mentioned, you can see how people adapt here.*
		Lack of effectiveness	Participant 9 shared her experience with a kidney transplant, stating: *I had a kidney transplant, but it was rejected after 8 years.*
		Lack of availability of medicine	Participant 9 also spoke about the challenges due to medicine shortages, saying: *We often can't find basic medicines here. Sometimes, we even have to provide the hospital with supplies like plasters because they run short. It's really tough. For instance, the pills I need for my blood pressure are sometimes unavailable at the hospital, forcing me to buy them elsewhere. I also have to purchase other items, like plasters, myself. Dialysis has become increasingly difficult these days.*
		Attitude towards medical procedures	Participant 2 expressed frustration with the dialysis process, preferring to continue with haemodialysis due to its fewer safety measures compared to peritoneal dialysis. He explained: *Today, a doctor asked me about my plan. I told him I did not want to do CAPD* [peritoneal dialysis]. *I'd rather continue with hemodialysis.* Participant 10 mentioned that he has little hope of being qualified to receive a kidney from the cadaver list. *They (the hospital) will not consider me because I am old and will give priority to younger patients* Participant 9 was not satisfied with the service provided by the dialysis unit. She expressed her concerns as: *I think they do this service just as a job. There is nothing beyond that. There are only a few who work with good motivation. They don't care about us. Not everybody, but the majority. You can see many doctors and nurses here in this unit. It requires a close relationship with the patient for them to feel comfortable. It helps with mental development too. Here, it is difficult, and they do not provide such a service. We are not getting satisfactory service.*
		Attitude towards transplantation	Participant 1 is considering dialysis due to difficulties in finding a suitable kidney donor. She expressed her concerns: *I don't have any family members who can donate a kidney to me. My blood group is O* + *. I'm hesitant to receive a kidney from someone else because I worry they might suffer health consequences because of it. I don't want anyone else to suffer on my account.* Participant 2 expressed that due to the difficulty and delay in finding a kidney, he decided to stay on dialysis for the rest of his life. *I made a decision that I would stay remaining short period of life by getting a treatment like dialysis. Now I am about 47 years old. So. after 50 years we are almost weak.* Participant 5 expressed that he would not receive any support from his siblings for kidney transplantation*.* *I cannot even imagine it. Who will give? I have two younger brothers. They did not come to my house, not even to see me.* Participant 6 stated that she does not want to put her children in trouble by searching for money for transplantation. *Anyhow, I will die. There is no need to put my children through the trouble of collecting money for donors. I do not want to be a burden to my family members*
		Attitude towards healthcare system	Participant 5 praised the hospital staff for their attentive and prompt assistance. She stated: *I think there's no need to complain. The hospital staff are commendable, always considering the patient's needs. Whenever I ask for help, they're readily available.* Participant 14 also expressed appreciation for the dedication of the medical staff and nurses, noting a contrast with the doctors in other wards. He said: *I feel everything is going smoothly. All the medical staff, particularly the nurses, are very dedicated. They differ from doctors in other wards in that we can ask nurses anything about the patient, and they always provide clear explanations.*

### 3.1 Theme 1: Standard of Living (Quality of Life)

The quality of life for individuals with CKD and their caregivers is impacted by three factors: work life, family life and everyday life.

#### 3.1.1 Work Life

The work lives of individuals with CKD and those undergoing dialysis treatment have been significantly impacted. Nearly all respondents discussed how their jobs were affected by these conditions. Some faced difficulties in continuing their jobs, while others had to stop working altogether, leading to early retirement. For example: participant 8 expressed: *While I am capable of working, the necessity of undergoing dialysis twice a week makes maintaining regular employment unfeasible. Taking two days off every week is not something I can afford.*

#### 3.1.2 Family Life

The impact of CKD on family dynamics varies depending on the patient's role within the family. Half of the patients surveyed (5 out of 10) spoke directly about the negative effects of the disease on family relationships. Primarily, the results highlighted how the disease impacts family members and intimate relationships. Unmarried individuals often feel isolated, receiving limited support from siblings due to their family commitments and economic constraints. The participants noted that some of their close relatives and friends distanced themselves after they fell ill, avoiding close relationships due to the disease. For example: participant 3 expressed: *I have two younger brothers who live nearby; one resides just half a kilometer away, and the other is even closer. Yet, despite the short distance of less than 1 kilometer, they haven*'*t visited me since I became ill. This is in stark contrast to our previously close relationship, as they have distanced themselves from me after my illness.*

#### 3.1.3 Everyday Life

The daily lives of participants are affected in various ways, including diet, financial issues, social isolation and the need for adaptation. Each of these factors significantly influences their day‐to‐day experiences and overall wellbeing. Both individuals undergoing dialysis and their caregivers highlighted the numerous challenges associated with the disease, particularly in terms of dietary restrictions. While striving to adhere to medical guidelines, many face financial constraints that hinder their ability to follow recommended dietary plans. For example: participant 1 explained: *To be honest, affording the diet recommended by the hospital is beyond my means. I simply don*'*t have the financial resources to purchase protein‐rich foods like meat, eggs, or milk. Currently, I weigh only 45 kg, and the hospital advises a daily intake of 40–50 grams of protein. Unfortunately, meeting this requirement is just not feasible for me.* A significant majority of individuals (9 out of 10) with CKD reported facing financial difficulties. These challenges include the high costs of travelling for treatments, medication expenses and managing basic living costs. Additionally, some individuals grappling with CKD have gradually acclimated to the progression of the disease, which is often linked with comorbid conditions like diabetes and hypertension.

### 3.2 Theme 2: Coping Strategies

The coping strategies employed by individuals with the disease and their caregivers include attitudes, psychosocial support, spirituality/religion and perception of the disease.

#### 3.2.1 Attitudes

The experience of chronic illness has elicited a range of attitudes among those affected. As noted earlier, individuals recounted numerous challenges resulting from their conditions, often leading to a sense of helplessness. These attitudes are intricately linked with the nature of the disease, influencing the patients' outlooks. For example: Participant 9 stated *I think they may believe they* (siblings and relatives) *will face troubles because of me. This is trouble. It is not cured. This condition is not going to be cured like a cold or a fever. It exists forever.* Remarkably, despite a majority expressing feelings of hopelessness and helplessness, with 4 out of 10 patients explicitly articulating this sentiment, some participants demonstrate a remarkable ability to focus on practical solutions. For instance, one individual emphasized the importance of adhering to the hospital's timetable, being meticulous about dietary habits and consistently taking prescribed medications. They follow a strict dietary regimen, abstain from unnecessary food and beverages like tea, and avoid meals outside their home. Additionally, they limit social interactions, including with friends visiting their house, as part of a dedicated effort to rebuild their lives in the face of these challenges. Because some patients strictly adhere to the health precautions recommended for individuals on dialysis. They were advised to minimize exposure to external people to prevent infections, which led them to avoid inviting friends to their homes. The primary aim here is to prioritize their health.

#### 3.2.2 Psychosocial Support

Despite facing numerous obstacles, individuals with CKD and their caregivers often receive support from various sources, including close relatives, friends and government programs. The data reveals that only 2 out of 10 individuals with CKD received government allowances. Furthermore, 7 out of 10 participants reported receiving satisfactory family support to cope with the disease. In addition, assistance from friends plays a vital role for some participants. For instance, Participants 4 and 7 mentioned that their friends were instrumental in helping them find a kidney for transplant. For example: Participant 4 mentioned: *I frequently meet with my friends and talk to them. They have been a great help to me, even in my search for a kidney.*

#### 3.2.3 Spirituality/Religion

Apart from relying on conventional healthcare systems, individuals coping with CKD and their caregivers frequently turn to their traditions for support. Religious beliefs, cultural practices and the personal meanings they assign to life provide considerable relief and serve as coping mechanisms to mitigate the stress associated with this challenging health condition. Many patients find solace in Buddhist teachings, which help ease the burdens of the chronic disease. They believe that focusing on religious thoughts can dissipate pain, ensuring a peaceful passing that will not burden others. Cultural practices aimed at alleviating mental and physical distress, such as Bodhi Puja—which involves worshiping the Bodhi tree and the presiding deity—and wearing protective pendants with mantras, are prevalent among these individuals.

The meaning of life for individuals with CKD and their caregivers is significantly influenced by religious and cultural perspectives, alongside their adherence to conventional medical treatments. According to Buddhism, letting go, suffering and the impermanent nature of life are emphasized. The relevant quotes related to the ‘meaning of life’ indicate that participants have had the shadow of Buddhism in their interpretation of life. For example: Participant 2 stated: *The solution is to practice letting go. Then we do not have problems.*

#### 3.2.4 Perception of the Disease

The majority of participants expressed surprise upon being diagnosed with CKD. Despite the disease's common association with diabetes and hypertension, there was a notable lack of awareness about it among the participants. This lack of knowledge led to confusion at the time of diagnosis. Furthermore, due to difficulties in accessing appropriate treatment, participants reported a diminished expectation of life. Additionally, none of the participants had a family history of CKD, leading them to ponder and attempt to reconcile their condition with the Buddhist concept of karma. They often view their suffering as a part of their destiny, a belief deeply ingrained in Buddhist culture. For example: Participant 1 expressed: *Since 2009, I*'*ve tried to protect myself. But perhaps this is my destiny.* This understanding intertwines the present suffering with notions of karma and destiny, thus shaping their perspectives on their current situation.

### 3.3 Theme 3: Medical Experience

This theme encompasses the processes of diagnosis, gaining knowledge and treatment as experienced by individuals with CKD.

#### 3.3.1 Process of Diagnosis

Participants with CKD reported various experiences with their diagnosis process. Some had been suffering from diabetes or hypertension for a long time, which eventually led to CKD. Others progressed to ESRD without prior awareness and suddenly began dialysis. The frequent testing required for dialysis and transplantation has caused feelings of insecurity among some participants. For example: Participant 13 explained: *The doctors informed me that they initiated dialysis due to my son*'*s elevated creatinine levels and revealed that his kidneys had been damaged. We were completely unaware of this disease.*

#### 3.3.2 Gaining Knowledge

Participants expressed a need for more comprehensive information about CKD. They felt that doctors often limited their engagement to prescribing medication, without providing in‐depth explanations or additional guidance. Moreover, several participants reported gaps in their understanding and knowledge of the disease, leading to confusion due to inconsistent or unclear information from various sources. For example: Participant 7 stated: *I*'*m not sure what this disease exactly is. Some say the kidneys are melting; others say they*'*re shrinking.*

#### 3.3.3 Treatment

The treatment of CKD involves various challenges, categorized into difficulties with travelling, treatment effectiveness, medication availability, procedural complexities, transplantation challenges and healthcare system interactions.

- Travel difficulties: Most participants reported significant difficulties travelling to receive treatments, citing high costs, long distances and physical discomfort as primary concerns.
- Treatment effectiveness and complexity: Many participants expressed disappointment with both the complexity of treatment procedures and their perceived lack of effectiveness.
- Medication shortage: Exacerbated by Sri Lanka's current economic crisis, a shortage of medication has become a significant burden for patients undergoing dialysis. Essential drugs, often unavailable locally and costing around RS 1600 ($5) each, have led patients to pool resources for joint purchases.
- Transplantation challenges: All 10 individuals with CKD faced considerable obstacles in securing a suitable kidney for transplantation. The primary challenge was finding a donor, particularly from within their families. Financial hurdles frequently led to seeking donors who expect monetary compensation, with some external donors demanding up to $6000. As an alternative, many patients are considering inclusion on the hospital's cadaver list for potential deceased donor kidneys.
- Community support: The community's support for these individuals was notable, with reports of friends initiating fundraising efforts to help finance their transplants. Hesitancy with non‐relative donors: Some individuals expressed reluctance to seek kidneys from non‐relatives, concerned about the potential harm or discomfort to the donors.
- Patient experience in dialysis units: While those receiving care in dialysis units expressed overall satisfaction, there was a desire for a more personable and engaging approach from medical staff. Patients long for interactions filled with humour and human connection, seeking temporary respite from the stress of their illness. For example: As participant 9 mentioned: *I think they do this service just as a job. There is nothing beyond that. There are only a few who work with good motivation. They don*'*t care about us. Not everybody, but the majority.*

## 4 Discussion

CKD represents a significant health challenge in Sri Lanka, marked by its high prevalence and extensive psychosocial impacts on patients and their caregivers. This qualitative study was conducted to explore the psychosocial experiences of these two groups. The content analysis revealed three main areas requiring intervention: standard of living (quality of life), coping strategies and medical experience. A particularly important factor identified was the power of tradition, encompassing general attitudes, beliefs and religious practices. These elements are integrated into the constructed model presented in Figure 1.

The study underscored the difficulties individuals face in managing daily activities and maintaining overall well‐being while living with CKD. Though this research was conducted in Sri Lanka, similar challenges are observed in other countries, as indicated by the analysis of Maguire, Hanly and Maguire [31]. They highlight the crucial role of social support, loneliness and psychological appraisals in sustaining wellbeing in a population‐based European sample, suggesting that positive appraisals may help individuals cope better with their conditions and mitigate daily life limitations. This aligns with findings from a systematic review by Roberti et al. [32], which encompassed 260 studies from 30 countries, involving 5115 patients and 1071 caregivers. This review emphasized socioeconomic status as a pivotal factor in the CKD experience, especially in advanced stages requiring renal replacement treatment. Challenges identified included underfunded healthcare, reliance on emergency care, risk of unemployment and insurance issues and financial strain. Patients often struggled with transportation to haemodialysis centres, particularly those in nonurban areas, or those with young children or limited resources. Uninsured or underinsured patients faced additional burdens, such as the need for fundraising. Post‐transplant patients contended with uncertainties regarding financial management and responsibilities. A common concern across the board was the lack of information about the disease, treatment options and side effects of immunosuppressants. Living with end‐stage kidney disease was described as highly burdensome, involving time‐consuming, invasive and exhausting tasks that affect all aspects of patients and caregivers.

Individuals with CKD and their caregivers utilize various coping strategies to manage the disease‐related challenges, as observed in our study. A systematic review by Shahin, Kennedy and Stupans [33] emphasized the significant influence of personal and cultural beliefs on medication adherence among patients with chronic diseases like hypertension and diabetes. This review highlighted the critical role of patients' individual perceptions and beliefs about their illnesses in adhering to prescribed medications. Similarly, Nair et al. [34] stressed the importance of considering patients' perspectives and beliefs when providing healthcare guidance and treatment. In our study, we noted that patients from diverse racial, ethnic and sociocultural backgrounds view advanced CKD as a substantial limitation in their lives, often experiencing a loss of control over the disease's progression. This leads to various psychological and emotional challenges, including anxiety about death, uncertainty regarding their prognosis and existential distress. To cope with these challenges, patients employ strategies such as accepting their condition, avoiding the implications of the disease, seeking solace in spiritual beliefs or finding support within religious communities. Notably, a preoccupation with and fear of death were identified as new factors contributing to psychological distress among individuals with CKD. Furthermore, several studies [35, 36, 37] have identified religion and acceptance of illness as important coping mechanisms. These findings, consistent with our study, suggest that individuals often rely on their religious beliefs and acceptance of their condition to cope with the psychosocial burden of CKD.

Our findings revealed that participants engage in meditation, a core teaching of Lord Buddha, and perform bodhi puja ceremonies at the bodhi tree. Some individuals wear jewellery (known as ‘Sura’ in Sinhala) with hopes of warding off negative influences. Moreover, many pray for recovery at religious sites, promising offerings upon regaining health. However, despite these cultural practices, all participants primarily trust mainstream medicine. They diligently follow medical guidelines despite facing considerable challenges. These results echo the findings of Wimalasena and Marks [38], indicating that while Sri Lanka is steeped in tradition, cultural practices are often habitual rather than reflective, lacking reliable guidance in social actions.

Interestingly, many participants in our study perceive CKD as a ‘karmic disease’, associating it with the concept of destiny. This viewpoint stems not just from religious beliefs but also from practical circumstances, as some participants link their condition to limited access to timely treatment, often due to poverty. Thus, the label of ‘karmic disease’ is partly influenced by their socioeconomic challenges. In their attempts to cope, several participants embrace the Buddhist teaching of the ‘impermanence of life’. This perspective is not solely a product of blind faith; rather, it reflects a coping mechanism influenced by their restricted access to healthcare resources. The inadequacy in accessing psychosocial support further encourages reliance on traditional health and life beliefs. Joshi [39] emphasizes the importance of addressing these subjective elements, including spiritual and religious belief systems, in enhancing the quality of life for ESRD patients. This highlights the need for a holistic approach to patient care that considers not only the medical but also the psychosocial and spiritual aspects of chronic illness management.

As demonstrated in our study, the medical experience of individuals with CKD plays a pivotal role in their psychosocial wellbeing, aligning with findings by Kalantar‐Zadeh et al. [40]. CKD requires continuous medical management, including a strict medication regimen, dietary limitations and, in some cases, the need for dialysis. These treatments often demand significant time and physical effort from patients. Our study highlighted that participants experienced a sense of ineffectiveness, lack of knowledge and frustration with the procedural aspects of their disease, leading to diminished perceptions of disease benefits. This was reflected in their minimal expectations regarding their condition's outcome. In contrast, a study by Rymon Lipińska and Nowicka‐Sauer [41] found a significant association between perceived benefits of the disease and more favourable illness perceptions among individuals with type 1 diabetes, indicating a critical link between disease benefit perception and mental wellbeing. This finding contrasts with the challenges faced by our study participants, who struggled with inadequate perceptions of their treatments. Furthermore, Hedayati et al. [42] identified a significant correlation between major depressive episodes and critical events such as the initiation of dialysis, hospitalization or death in patients with CKD. Their study noted that depression affects up to 20% of CKD patients even before dialysis initiation, underscoring the importance of addressing mental health issues to improve overall wellbeing in this population. Recommendations by Senanayake et al. [19] stress the need for periodic screening of all CKD patients in the rural districts of Sri Lanka for depression and psychological distress. They also suggest the necessity for policymakers to support an organized psychological health service, aimed at enhancing the mental wellbeing of individuals with CKD.

### 4.1 Research Strengths

- Comprehensive examination of both patients and caregivers: The study's approach included an in‐depth examination of the experiences of both patients with CKD and their caregivers, providing a holistic understanding of the disease's impact.
- Focused patient group: All participants were patients treated at a single centre in Sri Lanka, ensuring consistency in the treatment environment and healthcare delivery.
- Uniform disease severity: The study specifically focused on patients diagnosed with the same severity of CKD, all undergoing dialysis. This uniformity allows for more focused insights into this particular patient group.
- Rigorous medical diagnostics: The psychological examination of participants was preceded by a reliable medical diagnosis, ensuring that the psychological assessments were grounded in accurate and comprehensive medical understanding.

### 4.2 Study Limitations

This study faced several limitations that should be acknowledged. First, the sample size was small and included both patients with CKD and their caregivers. Given the qualitative nature of this research, the primary aim was not to generalize the findings but to provide an in‐depth understanding of the participants' experiences. Therefore, generalizability is not applicable to this type of study. Instead, the insights gained can serve as a basis for further research and potential interventions.

Additionally, the study focused on Sinhala Buddhist individuals, which may limit the relevance of the findings to other ethnic and religious groups in Sri Lanka. This specificity should be considered when interpreting the results and planning broader applications.

Data were collected retrospectively, requiring participants to recall their experiences at different stages of the illness. This approach may introduce recall bias, potentially affecting the accuracy of the reported experiences. While this limitation is inherent in qualitative research, the rich, detailed accounts provided by participants still offer valuable perspectives on the psychological and social challenges faced by patients with CKD and their caregivers in Sri Lanka.

Despite these limitations, the study provides important insights that can inform future research and interventions aimed at supporting patients with CKD and their caregivers.

### 4.3 Directions for Further Research

Future research should prioritize understanding the long‐term effects of CKD on the quality of life of both individuals with the disease and their caregivers. Longitudinal studies would be beneficial for assessing changes in psychosocial wellbeing over time and evaluating the effectiveness of coping strategies, including religious practices and acceptance of the illness. There is a significant need to explore the role of healthcare providers in supporting individuals with CKD and their caregivers. Investigating the experiences and perspectives of healthcare professionals can offer crucial insights into enhancing the medical experience and psychosocial support for those living with CKD.

We believe it would also be valuable to conduct studies with larger sample sizes, which would increase the robustness and generalizability of our results. This approach would enable a more comprehensive understanding of the psychological and social challenges faced by patients with CKD and their caregivers.

## 5 Conclusion

This study highlights the significant psychosocial impact of CKD on individuals and their caregivers in Sri Lanka. The findings underscore the necessity for comprehensive support systems that address the challenges encountered in everyday life, work life and family life. Future research should prioritize addressing the identified knowledge gaps to enhance our understanding of the psychosocial experiences of individuals with CKD and develop effective interventions to improve their quality of life, taking into account the specific influence of tradition and its power. A holistic approach to managing CKD is imperative, encompassing not only the physical aspects but also the emotional, psychological and social dimensions of the disease.

## Author Contributions

**Darshika Thejani Bulathwatta:** conceptualization, methodology, investigation, formal analysis, writing–original draft, data curation, visualization, writing–review and editing. **Agata Rudnik:** conceptualization, methodology, data curation, investigation, writing–original draft, writing–review and editing, formal analysis, visualization. **Mariola Bidzan:** conceptualization, supervision, project administration, methodology, writing–review and editing, formal analysis, investigation, funding acquisition.

## Ethics Statement

The study received ethical approval from the Ethical Review Committee of the Open University of Sri Lanka, under the application number ER/2022/007.

## Consent

Informed consent was obtained from all participants included in the study. The participants were briefed about the nature of the research, its purpose, procedures, potential risks and benefits. They were informed about their voluntary participation and the confidentiality of their identity. Permission was obtained to reproduce any copyrighted material from other sources used in this article.

## Conflicts of Interest

The authors declare no conflicts of interest.

## Supporting information

Supporting information.

## Data Availability

The data that support the findings of this study are available on request from the corresponding author. The data are not publicly available due to privacy or ethical restrictions.

## References

[1] A. Latame Komla , M. Raffray , C. Valérie , et al., “Women's Access to Kidney Transplantation in France: A Mixed Methods Research Protocol,” International Journal of Environmental Research and Public Health 19, no. 20 (2022): 13524, 10.3390/ijerph192013524. 36294104 PMC9603645

[2] N. H. Lameire , A. Levin , J. A. Kellum , et al., “Harmonizing Acute and Chronic Kidney Disease Definition and Classification: Report of a Kidney Disease: Improving Global Outcomes (KDIGO) Consensus Conference,” Kidney International 100, no. 3 (2021): 516–526, 10.1016/j.kint.2021.06.028. 34252450

[3] K. Kafle , S. Balasubramanya , and T. Horbulyk , “Prevalence of Chronic Kidney Disease in Sri Lanka: A Profile of Affected Districts Reliant on Groundwater,” Science of The Total Environment 694 (2019): 133767, 10.1016/j.scitotenv.2019.133767. 31756806

[4] A. C. Webster , E. V. Nagler , R. L. Morton , and P. Masson , “Chronic Kidney Disease,” Lancet 389, no. 10075 (2017): 1238–1252, 10.1016/s0140-6736(16)32064-5. 27887750

[5] G. T. Hernández and H. Nasri , “World Kidney Day 2014: Increasing Awareness of Chronic Kidney Disease and Aging,” Journal of Renal Injury Prevention 3 (2014): 3–4, 10.12861/jrip.2014.02. 25340154 PMC4206036

[6] C. P. Kovesdy , “Epidemiology of Chronic Kidney Disease: An Update 2022,” Kidney International Supplements 12, no. 1 (2022): 7–11, 10.1016/j.kisu.2021.11.003. 35529086 PMC9073222

[7] S. J. Senanayake , N. S. Gunawardena , P. Palihawadana , et al., “Out‐of‐Pocket Expenditure in Accessing Healthcare Services Among Chronic Kidney Disease Patients in Anuradhapura District,” Ceylon Medical Journal 62, no. 2 (2017): 100, 10.4038/cmj.v62i2.8475. 28697592

[8] O. A. Adejumo , I. O. Iyawe , A. A. Akinbodewa , O. S. Abolarin , and E. O. Alli , “Burden, Psychological Well‐Being and Quality of Life of Caregivers of End Stage Renal Disease Patients,” Ghana Medical Journal 53, no. 3 (2019): 190, 10.4314/gmj.v53i3.2. 31741490 PMC6842729

[9] S. K. Gunatilake , S. S. Samaratunga , and R. T. Rubasinghe , “Chronic Kidney Disease (CKD) in Sri Lanka—Current Research Evidence Justification: A Review,” Sabaragamuwa University Journal 13, no. 2 (2015): 31–58, 10.4038/suslj.v13i2.7680.

[10] S. J. Senanayake , “Chronic Kidney Disease in Sri Lanka: A Glimpse into Lives of the Affected,” Journal of the College of Community Physicians of Sri Lanka 24, no. 2 (2018): 56, 10.4038/jccpsl.v24i2.8158.

[11] G. Abraham , S. Varughese , T. Thandavan , et al., “Chronic Kidney Disease Hotspots in Developing Countries in South Asia,” Clinical Kidney Journal 9, no. 1 (2016): 135–141, 10.1093/ckj/sfv109. 26798474 PMC4720189

[12] M. V. A. R. Vithanage , R. M. L. Rathnayake , and D. J. Jagoda , “Factors Affecting the Prevalence of Chronic Kidney Disease Among Adult Population in Sri Lanka: With Special Reference to Badulla District,” Sri Lanka Journal of Social Sciences and Humanities 1, no. 2 (2021): 87–97, 10.4038/sljssh.v1i2.41.

[13] C. P. Andrade and R. C. Sesso , “Depression in Chronic Kidney Disease and Hemodialysis Patients,” Psychology 3, no. 11 (2012): 974–978, 10.4236/psych.2012.311146.

[14] T. M. Odette Dorcas , T. B. Youth , C. Atuhaire , G. Priebe , and S. N. Cumber , “Physiological and Psychosocial Stressors Among Hemodialysis Patients in the Buea Regional Hospital, Cameroon,” Pan African Medical Journal 30 (2018): 49, 10.11604/pamj.2018.30.49.15180. 30197740 PMC6125286

[15] Gerogianni Stavroula , “Psychological Aspects in Chronic Renal Failure,” Health Science Journal 8, no. 2 (2014): 205–214.

[16] S. Gerogianni , F. Babatsikou , G. Gerogianni , C. Koutis , M. Panagiotou , and E. Psimenou , “Social Life of Patients Undergoing Haemodialysis,” International Journal of Caring Sciences 9, no. 1 (2016): 122–134.

[17] M. V. Filgueiras de Assis and M. Angelo , “The Impact of Chronic Kidney Disease: Experiences of Patients and Relatives From the Extreme North of Brazil,” Investigación y Educación en Enfermería 36, no. 1 (2018): e02, 10.17533/udea.iee.v36n1e02. 29898341

[18] C. McKercher , K. Sanderson , and M. D. Jose , “Psychosocial Factors in People With Chronic Kidney Disease Prior to Renal Replacement Therapy,” Nephrology 18, no. 9 (2013): 585–591, 10.1111/nep.12138. 23876102

[19] S. Senanayake , N. Gunawardena , P. Palihawadana , C. Suraweera , R. Karunarathna , and P. Kumara , “Depression and Psychological Distress in Patients With Chronic Renal Failure: Prevalence and Associated Factors in a Rural District in Sri Lanka,” Journal of Psychosomatic Research 112 (2018): 25–31, 10.1016/j.jpsychores.2018.06.009. 30097132

[20] S. Senanayake , N. Gunawardena , P. Palihawadana , et al., “Health Related Quality of Life in Chronic Kidney Disease; A Descriptive Study in a Rural Sri Lankan Community Affected By Chronic Kidney Disease,” Health and Quality of Life Outcomes 18, no. 1 (2020): 106, 10.1186/s12955-020-01369-1. 32326945 PMC7178581

[21] C. Liyange , “Policy Aspects in Addressing Chronic Kidney Disease of an Unknown/Uncertain Etiology (CKDu),” Law and Society Trust 25 (2015), https://www.researchgate.net/publication/320622178.

[22] H. Ranasinghe and M. Ranasinghe , “Status, Gaps and Way Forward in Addressing the Chronic Kidney Disease Unidentified (CKDu) in Sri Lanka,” Journal of Environmental Professionals Sri Lanka 4, no. 2 (2015): 58, 10.4038/jepsl.v4i2.7863.

[23] C. Liyanage , “Chronic Kidney Disease of Uncertain Etiology in Sri Lanka: Curing Between Medicine and Traditional Culture,” Social Sciences 11, no. 1 (2022): 20, 10.3390/socsci11010020.

[24] W. Aruni , Sri Lanka Attorney‐at‐Law , Chronic Kidney Disease of Unknown Aetiology of Sri Lanka in Human Right Perspective: With Special Reference to National and International Human Rights Regime. *International Conference on Social Sciences*. Published online January 29, 2019, 10.17501/2357268x.2018.5101.

[25] J. H. Mills , “Margaret Jones. *Health Policy in Britain's Model Colony: Ceylon (1900–1948)*. (New Perspectives in South Asian History, 10.) xv + 305 pp., tables, illus., apps., bibls., index. Andhra Pradesh, India: Orient Longman, 2004. $33 (Cloth),” Isis 98, no. 2 (2007): 406–407, 10.1086/521476.

[26] A. Tong , P. Sainsbury , and J. Craig , “Consolidated Criteria for Reporting Qualitative Research (COREQ): A 32‐Item Checklist for Interviews and Focus Groups,” International Journal for Quality in Health Care 19, no. 6 (2007): 349–357, 10.1093/intqhc/mzm042. 17872937

[27] D. T. Bulathwatta , J. Borchet , A. Rudnik , and M. Bidzan , “Psychosocial Well‐Being Among Individuals With Chronic Kidney Disease Undergoing Hemodialysis Treatment and Their Caregivers: A Protocol of a Mixed Method Study in Sri Lanka and Poland,” Frontiers in Psychology 14 (2023): 1194991, 10.3389/fpsyg.2023.1194991. 38144983 PMC10740214

[28] H. J. H. Joffe , L. Yardley , and D. Marks , “Content and Thematic Analysis,” in Research Methods for Clinical and Health Psychology (London: SAGE Publications, Ltd., 2004), 56–68, 10.4135/9781849209793.n4.

[29] H. F. Hsieh and S. E. Shannon , “Three Approaches to Qualitative Content Analysis,” Qualitative Health Research 15, no. 9 (2005): 1277–1288, 10.1177/1049732305276687. 16204405

[30] S. Elo and H. Kyngäs , “The Qualitative Content Analysis Process,” Journal of Advanced Nursing 62, no. 1 (2018): 107–115, 10.1111/j.1365-2648.2007.04569.x. 18352969

[31] R. Maguire , P. Hanly , and P. Maguire , “Living Well With Chronic Illness: How Social Support, Loneliness and Psychological Appraisals Relate to Well‐Being in a Population‐Based European Sample,” Journal of Health Psychology 26, no. 10 (2019): 1494–1507, 10.1177/1359105319883923. 31647344

[32] J. Roberti , A. Cummings , M. Myall , et al., “Work of Being an Adult Patient With Chronic Kidney Disease: A Systematic Review of Qualitative Studies,” BMJ Open 8, no. 9 (September 2018): e023507, 10.1136/bmjopen-2018-023507. PMC6129107 30181188

[33] W. Shahin , G. A. Kennedy , and I. Stupans , “The Impact of Personal and Cultural Beliefs on Medication Adherence of Patients With Chronic Illnesses: A Systematic Review,” Patient Preference and Adherence 13, no. 1 (2019): 1019–1035, 10.2147/ppa.s212046. 31303749 PMC6611718

[34] D. Nair , K. Bonnet , M. G. Wild , et al., “Psychological Adaptation to Serious Illness: A Qualitative Study of Culturally Diverse Patients With Advanced Chronic Kidney Disease,” Journal of Pain and Symptom Management 61, no. 1 (2020): 32–41.e2, 10.1016/j.jpainsymman.2020.07.014. 32711122 PMC7770006

[35] C. Chatrung , S. Sorajjakool , and K. Amnatsatsue , “Wellness and Religious Coping Among Thai Individuals Living With Chronic Kidney Disease in Southern California,” Journal of Religion and Health 54, no. 6 (2014): 2198–2211, 10.1007/s10943-014-9958-4. 25300413

[36] A. M. Bravin , A. S. Trettene , L. G. M. Andrade , and R. C. Popim , “Benefits of Spirituality and/or Religiosity in Patients With Chronic Kidney Disease: An Integrative Review,” Revista Brasileira De Enfermagem 72, no. 2 (2019): 541–551, 10.1590/0034-7167-2018-0051. 31017220

[37] A. Burlacu , B. Artene , I. Nistor , et al., “Religiosity, Spirituality and Quality of Life of Dialysis Patients: A Systematic Review,” International Urology and Nephrology 51, no. 5 (2019): 839–850, 10.1007/s11255-019-02129-x. 30919258

[38] L. Wimalasena and A. Marks , “Habitus and Reflexivity in Tandem? Insights From Postcolonial Sri Lanka,” Sociological Review 67, no. 3 (2019): 518–535, 10.1177/0038026119825552.

[39] V. D. Joshi , “Quality of Life in End Stage Renal Disease Patients,” World Journal of Nephrology 3, no. 4 (2014): 308, 10.5527/wjn.v3.i4.308. 25374827 PMC4220366

[40] K. Kalantar‐Zadeh , T. H. Jafar , D. Nitsch , B. L. Neuen , and V. Perkovic , “Chronic Kidney Disease,” Lancet 398, no. 10302 (2021): 786–802, 10.1016/s0140-6736(21)00519-5. 34175022

[41] W. Rymon Lipińska and K. Nowicka‐Sauer , “Illness Perception and Perceived Benefits of Illness Among Persons With Type 1 Diabetes,” Health Psychology Report 11, no. 3 (2022): 200–212, 10.5114/hpr/153999. 38084261 PMC10670789

[42] S. S. Hedayati , “Association Between Major Depressive Episodes in Patients With Chronic Kidney Disease and Initiation of Dialysis, Hospitalization, or Death,” JAMA 303, no. 19 (2010): 1946, 10.1001/jama.2010.619. 20483971 PMC3217259

